# Maternal and Neonatal Levels of Perfluoroalkyl Substances in Relation to Gestational Weight Gain

**DOI:** 10.3390/ijerph13010146

**Published:** 2016-01-20

**Authors:** Jillian Ashley-Martin, Linda Dodds, Tye E. Arbuckle, Anne-Sophie Morisset, Mandy Fisher, Maryse F. Bouchard, Gabriel D. Shapiro, Adrienne S. Ettinger, Patricia Monnier, Renee Dallaire, Shayne Taback, William Fraser

**Affiliations:** 1 Perinatal Epidemiology Research Unit, IWK Health Centre, Halifax, NS B3K 6R8, Canada; jillian.ashley-martin@dal.ca; 2 Department of Obstetrics and Gynecology, Dalhousie University, Halifax, NS B3K 6R8, Canada; 3 Health Canada, Ottawa, ON K1A 0K9, Canada; Tye.Arbuckle@hc-sc.gc.ca (T.E.A.); mandy.fisher@hc-sc.gc.ca (M.F.); 4 Sainte Justine Hospital Research Center, University of Montreal, Montreal, PQ H3T 1C5, Canada; anne-sophie.morisset@fsaa.ulaval.ca; 5 Department of Environmental and Occupational Health, University of Montreal, Montreal, PQ H3T 1A8, Canada; maryse.bouchard@umontreal.ca; 6 Department of Epidemiology, Biostatistics and Occupational Health, McGill University, Montreal, PQ H3A 1A2, Canada; gabriel.shapiro@mcgill.ca; 7 Departmentof Nutritional Sciences, University of Michigan School of Public Health, Ann Arbor, MI 48109, USA; adrienne.ettinger@gmail.com; 8 Department of Obstetrics and Gynecology, McGill University, Montreal, PQ H3A 1A1, Canada; monnier.patricia@gmail.com; 9 Faculty of Medicine, Laval University, Quebec City, PQ G1V 0A6, Canada; renee.dallaire@crchudequebec.ulaval.ca; 10 Departments of Pediatrics and Child Health, University of Manitoba, Winnipeg, MB R3E 3P4, Canada; tabacksp@cc.umanitoba.ca; 11 Departments of Obstetrics and Gynecology, University of Sherbrooke, Sherbrooke, PQ J1H 5N4, Canada; William.Fraser@usherbrooke.ca

**Keywords:** perfluoroalkyl substances, gestational weight gain, environmental contaminants, birth cohort

## Abstract

Perfluoroalkyl substances (PFASs) are ubiquitous, persistent pollutants widely used in the production of common household and consumer goods. There is a limited body of literature suggesting that these chemicals may alter metabolic pathways and growth trajectories. The relationship between prenatal exposures to these chemicals and gestational weight gain (GWG) has received limited attention. One objective was to analyze the associations among maternal plasma levels of three common perfluoroalkyl substances (perfluorooctanoate (PFOA), perfluorooctanesulfonate (PFOS), perfluorohexanesulfanoate (PFHxS)) and GWG. Additionally, we explored whether GWG was associated with cord blood PFAS levels. This study utilized data collected in the Maternal-Infant Research on Environmental Chemicals (MIREC) Study, a trans-Canada cohort study of 2001 pregnant women. Our analysis quantified associations between (1) maternal PFAS concentrations and GWG and (2) GWG and cord blood PFAS concentrations. Maternal PFOS concentrations were positively associated with GWG (*β* = 0.39 95% CI: 0.02, 0.75). Interquartile increases in GWG were significantly associated with elevated cord blood PFOA (OR = 1.33; 95% CI: 1.13 to 1.56) and PFOS (OR = 1.20; 95% CI: 1.03 to 1.40) concentrations. No statistically significant associations were observed between GWG and either measure of PFHxS. These findings warrant elucidation of the potential underlying mechanisms.

## 1. Introduction

Perfluoroalkyl substances (PFASs) are ubiquitous, globally persistent pollutants that are widely used as surfactants in the production of common household and consumer goods including cookware, clothing, and food packaging [1]. These chemicals are characterized by their stability, persistence and ability to repel oil and water [1]. Despite the fact that common PFASs, such as perfluorooctanoate (PFOA), perfluorooctanesulfonate (PFOS), and perfluorohexanesulfanoate (PFHxS) are being phased out [2,3], exposure in humans remains ubiquitous. The majority of North American women have detectable concentrations of PFOA, PFOS, and PFHxS [4,5,6]. Moreover, these PFASs have the capacity to cross the placenta, hence directly exposing the fetus [4,7].

Human and animal data suggest that PFASs may disrupt endocrine signaling [8,9] and alter adipocyte profiles [10] and expression of adipocyte genes [11]. Moreover, a review of the human and animal evidence concluded that *in utero* exposure to PFOA is associated with reduced fetal growth [12,13]. Further exploration of growth- and weight-related effects of PFAS exposure is warranted, especially for gestational weight gain (GWG). Pregnancy is a time of enhanced susceptibility to the potential adverse effects of environmental contaminants due to physiological and behavioural changes [14]. Furthermore, GWG is a critical predictor of multiple maternal and neonatal outcomes, such as increased birth weight and weight gain retention in the mother, for which there is limited information regarding potential environmental-related predictors [15]. While several studies have reported that lower GWG is associated with neonatal levels of persistent organic pollutants (POPs) [16,17,18], there has not, to our knowledge, been a similar investigation regarding the associations between PFASs and GWG. Moreover, the study by Vafeiadi *et al*. [17], reported that the inverse association between maternal persistent organic pollutant exposure and birth weight was observed among women with inadequate or excess GWG but not among women with adequate GWG. Though sufficient understanding of the impact of GWG on placental transfer of environmental contaminants is lacking, these limited studies suggest a possible relation between GWG and neonatal chemical burden. This investigation is worthy as it will build scientific understanding of how maternal physiological changes during pregnancy can impact neonatal environmental contaminant burden.

Given the lack of information regarding environmental predictors of GWG and the demonstrated role of GWG as a determinant of fetal outcomes, we determined the association between 1st trimester PFAS plasma levels and GWG. Additionally, we explored whether GWG was associated with cord blood PFAS levels. Secondary objectives were to look at effect modification by pre-pregnancy BMI and infant sex in these relations. This objective, which was to determine the nature of the relation between GWG and neonatal PFAS concentrations, was informed by the aforementioned previous literature regarding associations between GWG and neonatal POP concentrations [16,17,18].

## 2. Materials and Method

### 2.1. Study Population

Data and biospecimens were obtained from the Maternal-Infant Research on Environmental Chemicals (MIREC) Study, a trans-Canada cohort study of 2001 pregnant women. Study participants were recruited from 10 Canadian cities between 2008 and 2011. Briefly, women were eligible for inclusion if they were <14 weeks gestation at time of recruitment, ≥18 years of age, able to communicate in French or English, and planning to deliver at a local hospital. Women with a serious medical conditions or known fetal or chromosomal anomalies in the current pregnancy were excluded from the study [19]. The sample in the present investigation included mothers who had a singleton live birth, delivered at ≥34 weeks, and had no missing data on gestational weight gain or the chemicals of interest. All subjects gave their informed consent for inclusion before they participated in the study. The study was conducted in accordance with the Declaration of Helsinki, and the protocol was approved by the ethic review boards at Health Canada (REB 2011-0035), St. Justine’s Hospital (Montreal, QC, Canada) (REB #: 3440), and the IWK Health Centre (Halifax, NS, Canada) (REB #: 1008457).

### 2.2. Gestational Weight Gain Assessment

GWG was calculated based on a rate of weekly gain during the second and third trimesters. This approach does not rely on the last measured weight prior to delivery to be an accurate representation of delivery weight. For approximately ten percent of MIREC study participants, the last measured weight prior to delivery was four or more weeks prior to the delivery date and, therefore, not a reliable proxy for delivery weight. GWG rates were calculated based on the following formula:

$$\mathrm{GWG}\;\mathrm{Rate}=\frac{\mathrm{last}\;\mathrm{weight}\;\mathrm{measured}\;\mathrm{prior}\;\mathrm{to}\;\mathrm{delivery}\;(\mathrm{kg})-1\mathrm{st}\;\mathrm{trimester}\;\mathrm{visit}\;\mathrm{weight}\;(\mathrm{kg})}{\#\;\mathrm{weeks}\;\mathrm{between}\;\mathrm{last}\;\mathrm{measured}\;\mathrm{weight}\;\mathrm{prior}\;\mathrm{to}\;\mathrm{delivery}\;\mathrm{and}\;1\mathrm{st}\;\mathrm{trimester}\;\mathrm{visit}\;\mathrm{weight}}$$

GWG rate was evaluated according to the U.S. Institute of Medicine (IOM) gestational weight gain guidelines for 2nd and 3rd trimester weekly GWG rates [15] as has been done previously [20,21]. These guidelines provide ranges of recommended total amounts of GWG based on a women’s pre-pregnancy BMI. Considering that both inadequate and excess amounts of GWG are associated with potential adverse outcomes, the guidelines provide ranges to indicate whether a woman’s weight gain is below, within, or above the recommended amount of GWG. Total GWG was estimated using the following formula (where 27 represents the number of weeks in the second and third trimester) [20]: Total GWG (kg) = Early pregnancy weight change (kg) + (27 weeks × GWG rate (kg/week)).

Early pregnancy weight change was based on the difference between 1st trimester weight and self-reported pre-pregnancy weight. If the first trimester visit occurred prior to 12 weeks or if the relevant weight values were missing, then a default value of 2 kg was used as the value for early pregnancy weight gain [15]. For women with missing GWG data based on measured weights, we used a self-reported measure of GWG based on study participant’s response to the question (administered between 3 and 8 weeks post-delivery): “Approximately how much weight did you gain during this pregnancy”? We excluded values with missing data for weight or date, or if weights were implausible (<−5, >50 kg), or if there were less than four weeks between the last measured weight and the 1st trimester weight.

### 2.3. Perfluoroalkyl Substance Analysis

Chemical analysis of samples was carried out at the Laboratoire de Toxicologie, Institut National de Santé Publique du Québec (Québec, QC, Canada), accredited by the Standards Council of Canada. Perfluoroalkyl substances were measured in 1st trimester and venous cord blood plasma using a Waters Acquity UPLC-MS-MS operated in the MRM mode with an electrospray ion source in negative mode. The three perfluoroalkyl substances measured in maternal plasma and cord blood in MIREC study participants were PFOA, PFOS, and PFHxS, with limits of detection (LODs) ranging from 0.1 to 0.3 μg/L.

### 2.4. Statistical Analysis

PFASs were transformed by log base 2 prior to use as a continuous variable to normalize the distribution and to facilitate calculation of parameter estimates per a doubling of concentration. All maternal samples with values below the limit of detection were assigned a value of LOD/2. Due to the high percentages of cord blood samples with PFAS values below the LOD, particularly for PFOS and PFHxS, these chemicals were dichotomized at the limit of detection to represent detectable *vs.* non-detectable exposure concentrations. Spearman correlation coefficients were calculated among PFAS measurements. Due to the potential for biased results, correlation coefficients were only calculated for chemicals with more than 25% of values above the LOD.

Information on demographics, lifestyle, and reproductive history was obtained from chart review and questionnaires administered throughout pregnancy. Covariates were chosen for inclusion in multivariable analysis based on literature regarding predictors of GWG [15] and maternal or neonatal PFAS levels [22,23]. Parity, maternal age, income, and pre-pregnancy BMI were identified as predictors of both GWG [15,24,25] and maternal PFAS levels [22,23,26] and included in the adjusted models. Although smoking has been reported to be associated with GWG [15] and PFAS concentrations [22], we determined that smoking was not a confounder in the present study and was, therefore, not included in multivariate models. We based this decision on the lack of statistically significant associations between smoking and GWG, and between smoking and PFAS concentrations. This lack of confounding may be driven by the low smoking rate among MIREC study participants. Parity and BMI were identified as potential predictors of both GWG and cord blood PFAS levels [27] and, therefore, included in this model.

Restricted cubic spline models were used to assess the linearity of PFAS-GWG relations. As none of the models deviated from linearity, linear regression was used to assess the association between each log_2_ transformed PFAS and a continuous measure of GWG.

There are multiple possible mechanisms underlying the relations among BMI, PFAS, and GWG. For example, PFAS may alter adiposity and, therefore, influence BMI. On other hand, women with higher BMI may have more sources of dietary exposure to PFASs and, therefore, higher contaminant concentrations. We tested for effect modification by evaluating the *p*-value of the BMI-exposure interaction term and by stratifying by pre-pregnancy BMI. Stratification allowed us to examine the relation between PFAS and GWG independent of BMI. Due to the small number of women with an underweight pre-pregnancy BMI, these women were combined with those with a normal pre-pregnancy BMI in the stratified analyses.

Logistic regression models, adjusted for age and pre-pregnancy BMI, were used to measure the association between a continuous measure of GWG and a dichotomous measure of cord blood PFAS levels. Due to the high percentage of cord blood samples with PFOS (51.8%) and PFHxS (62.0%) concentrations below the LOD, these chemicals were dichotomized at their respective LOD. As only 11% of PFOA samples were below the LOD, this variable was dichotomized at the median. The cord blood PFAS analyses were stratified by infant sex as PFOA has been shown to have a longer half-life and slower renal clearance in males [28]. Odds ratios based on a per-unit (1 kg) and interquartile range (IQR) (7 kg) increase in GWG were calculated.

All statistical analyses were conducted using SAS v. 9.3 (SAS Institute Inc., Cary, NC, USA).

## 3. Results and Discussion

Of the 1826 women with singleton, live births, and gestational age >34 weeks, 69 were missing GWG data. Of the remaining 1757 women with GWG data who met the study inclusion criteria, 34 participants were missing first trimester PFAS data and 456 were missing cord blood PFAS data. The analytical samples thus comprised 1723 participants with first trimester maternal exposure data and 1301 participants with cord blood exposure data.

Study population characteristics are shown in Table 1. The majority of women were of normal pre-pregnancy BMI, gained in excess of the U.S. IOM gestational weight gain guidelines, had a household income greater than $50,000, and were non-smokers. Descriptive statistics for key demographic characteristics were compared between the analytic sample and the whole MIREC cohort. The rate of current smokers was slightly lower (5.1%) in the analytical sample than the overall cohort (5.5%). There were no notable differences in maternal age at delivery or pre-pregnancy BMI between the analytical sample and the overall cohort.

Median maternal plasma PFAS concentrations were higher than those in cord blood (Table 2). The percentage of maternal plasma samples with detectable values of PFOA, PFOS, and PFHxS was 99.8%, 99.8%, and 95.7%, respectively. The percentage of cord blood plasma samples with detectable values of PFOA, PFOS, and PFHxS was 89.1%, 48.2%, and 38.0%, respectively. The Spearman correlation coefficient among chemicals varied from 0.34 (between cord blood PFOA and maternal PFHxS) to 0.68 (between maternal and cord blood levels of PFOA) (all *p*-values < 0.05).

This association between 1st trimester PFAS concentrations and total GWG did not vary according to pre-pregnancy BMI (all *p*-values for the BMI **x**PFAS product terms >0.1) (Table 3). In order to present estimates independent of pre-pregnancy BMI, all results are presented by strata of BMI. Among underweight/normal and obese women, each PFAS was positively associated with modest increases in GWG though the association was only statistically significant in the PFOS-GWG model among underweight/normal and normal women. Doubling PFOS concentrations was associated with a 0.39 (95% CI: 0.02, 0.75) kg increase in GWG in the underweight/normal pre-pregnancy BMI subgroup. Among overweight women, the association was positive for PFOA and inverse for PFOS and PFHxS though the null value was included in all confidence intervals.

In the analysis using continuous GWG as the independent variable, GWG was significantly associated with elevated odds of high cord blood PFOA and PFOS concentrations (Table 4). An interquartile increase in GWG (7 kg) was associated with 33% increase in odds of high cord blood PFOA levels (95% CI: 1.13, 1.56). Associations were similar among male (OR = 1.40 95% CI: 1.12, 1.75) and female infants (OR = 1.26 95% CI: 1.00, 1.59) (*p*-value interaction term = 0.76). An IQR increase in GWG exposure was also associated with significantly increased odds of high PFOS exposure (OR = 1.20 95% CI: 1.03, 1.40) with similar results among males and females.

**Table 1:** Characteristics of study participants, MIREC study, 2008–2011 (*N* = 1723) **^a^**.

Characteristic	25%	Median	75%		Range
Maternal age (y)	29.0	33.0	37.0		18.0–49.0
Gestational age (wks)	38.0	39.0	40.0		34.0–42.0
Birthweight (g)	3170	3473.0	3785		1630–5620
Gestational weight gain (kg)	11.7	15.2	18.7		−3.8–44.5
		**N**		**%**	
Pre-pregnancy BMI, kg/m^2^ **^b^**					
Underweight (<18.5)		44		2.7	
Normal (18.5 to 24.9)		993		61.6	
Overweight (25 to 29.9)		348		21.6	
Obese (≥30)		228		14.1	
Gestational weight gain **^c^**					
Below Recommendations		278		17.8	
Within Recommendations		403		25.8	
Above Recommendations		883		56.5	
Household income ($CAD)					
≤30,000		128		7.7	
30,001–50,000		158		9.6	
50,001–100,000		683		41.4	
>100,000		679		41.2	
Mode of delivery					
Vaginal		1246		72.3	
Caesarean		477		27.7	
Maternal smoking					
Never or quit before pregnancy		1517		88.0	
Quit during pregnancy		119		6.9	
Current		87		5.1	
Parity					
0		742		43.1	
1		696		40.4	
≥2		283		16.4	
Infant sex					
Male		902		52.4	
Female		821		47.7	

Abbreviations: MIREC, Maternal-Infant Research on Environmental Chemicals; IQR, interquartile range; PFOA, perfluorooctanoate; PFOS, perfluorooctanesulfonate; PFHxS, perfluorohexanesulfanoate; **^a^** subtotals may not equal sample size due to missing covariate data; **^b^** According to the World Health Organization; **^c^** According to the U.S. IOM guidelines [15].

**Table 2:** Descriptive statistics of first trimester maternal and cord blood plasma perfluoroalkyl substance levels (ng/mL) **^a^**.

Perfluoroalkyl Substance	First Trimester (*N* = 1723)				Cord Blood (*N* = 1301)			
	25%	Median	75%	Range	25%	Median	75%	Range
Perfluorooctanoate (PFOA)	1.10	1.70	2.40	LOD–16	0.22	0.39	0.61	LOD–5.60
Perfluorooctanesulfonate (PFOS)	3.30	4.60	6.80	LOD–36	0.15	0.15	0.77	LOD–5.80
Perfluorohexanesulfanoate (PFHxS)	0.66	1.00	1.60	LOD–40	0.10	0.10	0.29	LOD–1.9

**^a^** First trimester LODs = 0.1 (PFOA), 0.3 (PFOS), 0.3 (PFHxS). Cord blood LODs = 0.3 (PFOA), 0.3 (PFOS), 0.3 (PFHxS) (all ng/mL).

**Table 3:** Multivariate linear regression of first trimester log_2_ perfluoroalkyl substance concentrations (ng/mL) and total GWG (parameter coefficients, 95% CI) stratified by pre-pregnancy BMI.

Perfluoroalkyl Substance	Underweight /Normal ^a^ *N* = 1036	Overweight ^a^ *N* = 345	Obese ^a^ *N* = 228
	*β* (95% CI)	*β* (95% CI)	*β* (95% CI)
PFOA	0.38 (−0.03, 0.79)	0.58 (−0.26, 1.42)	0.38 (−0.81, 1.56)
PFOS	0.39 (0.02, 0.75)	−0.08 (−0.94, 0.78)	0.10 (−1.07, 1.29)
PFHxS	0.12 (−0.14, 0.38)	−0.12 (−0.73, 0.48)	0.50 (−0.32, 1.31)

**^a^** Adjusted for age, income, and parity.

**Table 4:** Logistic regression analysis of association between GWG (continuous) and binary measures of cord blood perfluoroalkyl substances concentrations per 1 kg and interquartile range increase in GWG.

GWG Increase ^a^	N	PFOA >0.39 ng/mL ^c^	PFOS >0.30 ng/mL ^d^	PFHxS >0.30 ng/mL ^d^
		OR (95% CI)	OR (95% CI)	OR (95% CI)
Total	1223			
1 kg		1.04 (1.02, 1.06)	1.03 (1.00, 1.05)	1.01 (0.99, 1.03)
IQR **^b^**		1.33 (1.13, 1.56)	1.20 (1.03, 1.40)	1.07 (0.91, 1.25)
Males	650			
1 kg		1.05 (1.02, 1.08)	1.02 (0.99, 1.05)	1.01 (0.98, 1.04)
IQR **^b^**		1.40 (1.12, 1.75)	1.20 (0.93, 1.41)	1.09 (0.88, 1.35)
Females	573			
1 kg		1.01 (1.00, 1.06)	1.03 (1.00, 1.07)	1.00 (0.97, 1.03)
IQR **^b^**		1.26 (1.00, 1.59)	1.28 (1.02, 1.60)	1.02 (0.81, 1.29)

**^a^** Adjusted for parity, pre-pregnancy BMI; **^b^** Interquartile range (IQR) increase = 7 kg; **^c^** dichotomized at median; **^d^** dichotomized at LOD.

### Discussion

In this cohort of Canadian women and their newborns, a doubling of PFOS concentrations was associated with modest, statistically significant increases in GWG among women in the underweight or normal pre-pregnancy BMI category but not the overweight or obese pre-pregnancy BMI category. It is possible that the lack of statistical significance in the overweight and obese categories was influenced by the lower sample size. For example, the magnitude of association between PFOA and GWG is the same among underweight and obese women yet the confidence interval is notably wider among the smaller subgroup of obese women. We also observed that 1 kg and IQR (7 kg) increases in GWG were associated with statistically significantly elevated concentrations of cord blood PFOA and PFOS. There was no observed effect modification by infant sex in the relationship between GWG and cord blood PFAS concentrations. Exposure concentrations in the MIREC study were comparable to a representative sample of the Canadian population. Median PFAS concentrations in women ages 20–39 in the Canadian Health Measures Study (median PFOS = 6.4 ug/L, PFOA = 2.1 ug/L, PFHxS = 1.2 ug/L) were comparable to MIREC study participants (median PFOS = 4.6 ug/L, PFOA = 1.7 ug/L, PFHxS = 1.0 ug/L) [6].

A limited number of epidemiological and experimental studies provide some insight into the physiological relations between PFAS exposure and metabolic and hormonal pathways. One birth cohort study reported that *in utero* PFOA exposure was associated with obesity among the offspring at age 20 [10]. Low-dose *in utero* PFOA exposure has been reportedly associated with mid-life increases in leptin and insulin in an animal model [8]. A mouse cell study reported that all three PFASs measured in this study were associated with increased expression of genes, such as the leptin gene, involved in lipid metabolism and adipocyte differentiation [11]. These findings provide some biological plausibility for a positive association between PFAS exposure and GWG. Among adults, a limited number of studies have examined the relations between PFAS concentrations and metabolic function. For example, a cross-sectional analysis of the Canadian Health Measures Study reported that blood PFHxS concentrations, but not PFOS or PFOA, were positively associated with total cholesterol levels in adults [29]. Another study among pregnant women reported that maternal PFAS concentrations were associated with higher levels of thyroid hormones [30].

However, PFAS exposure has also been shown to induce peroxisome proliferator-activated receptor (PPAR) pathways [8]. PPAR pathway activation may minimize obesity related inflammation and have an anti-obesogenic effect [31]. This potential mechanism provides biological plausibility for the previously observed inverse association between PFOA exposure and birth weight [12,13]. In sum, PFAS exposure may operate through multiple physiological pathways involved in hormonal and metabolic homeostasis. Moreover, these multiple pathways may operate in opposing manners. It is not possible, based on our data, to determine which, if any, of these mechanisms underlie the observed results. Further experimental work that builds upon current understanding of the adiposity related effects of PFAS [32] and that attempts to determine how placental transfer of PFAS varies according to GWG and BMI is necessary to define biological mechanisms underlying the observed results.

Due to the ubiquity of exposure and multiple pathways for contact with PFASs, it is also difficult to determine the relative contribution of different exposure sources to an individual’s body burden [33]. Diet, particularly ingestion of animal fats, meat, and snack foods (e.g., microwave popcorn), is an established and primary source of PFAS exposure [33,34]. Ingestion of contaminated food may result in simultaneous increases in caloric intake, leading to gestational weight gain and its association with maternal PFOA levels. We attempted to preserve a temporal relation between maternal exposure and GWG by using a first trimester measure of exposure. However, due to their long half-life, first trimester measures are correlated with levels throughout pregnancy [26,35]. Thus, we cannot definitively state whether maternal PFOA concentrations are a determinant of GWG or a consequence of the ingestion of contaminated food that may elevate both PFOA levels and GWG. Considering that specific sources of exposure cannot be ascertained based on maternal and fetal blood concentrations, it is difficult to determine whether maternal ingestion patterns (consumption of PFAS contaminated food via food packaging, cookware, or processing) contributed to the observed relationships. The observed associations between GWG and neonatal PFOA exposure may be driven by the high correlation (*r* = 0.68) between maternal and cord blood measures. If women with excess GWG have high PFOA levels, the relation between GWG and cord blood PFOA levels may be explained by the distribution of maternal contaminants into the fetal compartment.

Our study has several strengths, notably the relatively large sample size and availability of PFAS data in both mothers and cord blood. We were able to control for key covariates and had a study population recruited from 10 different cities across Canada. Interpretation of our results warrants consideration of three primary limitations. First, as previously noted, is the inability to disentangle the potential influence of an unmeasured confounder, namely dietary patterns, on the relation between PFOA and GWG. Second, it is possible that our results were confounded by maternal changes in plasma volume. As plasma volume increases throughout pregnancy [36], levels of contaminants may be diluted. We anticipate this potential influence to be minimal because maternal plasma PFAS concentrations were measured during the first trimester, prior to the time when maximal changes in plasma volume occur [36,37]. Any potential influence of plasma volume dilution would have likely resulted in negative confounding and an underestimate of the true associations between maternal PFAS concentrations and GWG. Cord blood PFASs concentrations are largely insulated from plasma volume changes [36], and, therefore, also unlikely to be influenced by plasma dilution. Third, we did not have the capacity to adjust for other physiological characteristics, namely glomerular filtration rate [38], that may confound the observed associations. In addition, due to the exploratory nature of this research, our objective was to examine the associations with individual chemicals. We did not attempt to examine potential synergy among chemicals or cumulative exposure. Fourth, the lack of statistical significance in the overweight and obese categories may have been influenced by the low sample size in these subgroups. Replication in a study with a larger sample size would be necessary to rule out type 2 error in the null association between maternal PFAS concentrations and gestational weight gain.

## 4. Conclusions

Our analysis of the associations among maternal, neonatal PFAS exposure levels and GWG raises interesting questions regarding the complex pathways between maternal and neonatal chemical burdens and GWG that warrant further investigation. In particular, the observed associations between excess gestational weight gain and elevated PFOA and PFOS cord blood concentrations suggests that maternal physiological changes during pregnancy may influence neonatal chemical burden. Due to the unique nature of the MIREC study population, replication in populations of differing socioeconomic classes, ethnicities, and health profiles would heighten the generalizability of the present findings.
